# Food Prices and Consumer Demand: Differences across Income Levels and Ethnic Groups

**DOI:** 10.1371/journal.pone.0075934

**Published:** 2013-10-02

**Authors:** Cliona Ni Mhurchu, Helen Eyles, Chris Schilling, Qing Yang, William Kaye-Blake, Murat Genç, Tony Blakely

**Affiliations:** 1 National Institute for Health Innovation, The University of Auckland, Auckland, New Zealand; 2 New Zealand Institute for Economic Research, Wellington, New Zealand; 3 Department of Economics, University of Otago, Dunedin, New Zealand; 4 Department of Public Health, University of Otago, Wellington, New Zealand; Old Dominion University, United States of America

## Abstract

**Background:**

Targeted food pricing policies may improve population diets. To assess their effects on inequalities, it is important to determine responsiveness to price changes across income levels and ethnic groups.

**Objective:**

Our goal was to estimate price elasticity (PE) values for major commonly consumed food groups in New Zealand, by income and ethnicity. PE values represent percentage change in demand associated with 1% change in price of that good (own-PE) or another good (cross-PE).

**Design:**

We used food expenditure data from national household economic surveys in 2007/08 and 2009/10 and Food Price Index data from 2007 and 2010. Adopting an Almost Ideal Demand System approach, own-PE and cross-PE estimates were derived for 24 food categories, household income quintiles, and two ethnic groups (Māori and non-Māori).

**Results:**

Own-PE estimates (with two exceptions) ranged from −0.44 to −1.78. Cross-PE estimates were generally small; only 31% of absolute values were greater than 0.10. Excluding the outlier ‘energy drinks’, nine of 23 food groups had significantly stronger own-PEs for the lowest versus highest income quintiles (average regression-based difference across food groups −0.30 (95% CI −0.62 to 0.02)). Six own-PEs were significantly stronger among Māori; the average difference for Māori: non-Māori across food groups was −0.26 (95% CI −0.52 to 0.00).

**Conclusions:**

Food pricing policies have potential to improve population diets. The greater sensitivity of low-income households and Māori to price changes suggests the beneficial effects of such policies on health would be greatest for these groups.

## Introduction

Good nutrition is essential for health [1]. A number of leading bodies have advocated fiscal policies to improve the nutritional quality of diets consumed, raise revenue to support population health interventions, and send a clear message to consumers about which foods are healthier [2], [3]. In recent times Denmark introduced a saturated fat tax (now revoked), Hungary a “junk food” tax, and France a tax on soft drinks. In the United States, 40 states implement small sales taxes on sweetened drinks. Recent reviews suggest such health-related food taxes and subsidies are likely to shift consumption in the desired direction and improve health [4]–[7].

The impact of changes in food prices on consumer demand is estimated using price elasticities. Price elasticity (PE) of demand measures the percentage change in purchased quantity or demand with a 1% change in price [8]. Own-PE refers to changes in demand for a food due to changes in its own price; cross-PE refers to changes in demand for a food in response to price changes in another food [8]. Because food is a necessity, it is generally believed that demand for food is relatively price ‘inelastic’, i.e. changes in price have a relatively small effect on the quantity purchased.

One concern regarding food taxes and subsidies is that unintended compensatory or displacement impacts could undermine their health objectives. For example, a subsidy on fruit and vegetables should increase purchases of fruit and vegetables (own-PE). However, consumers might also purchase more of other foods high in saturated fat and sodium because of cross-PE effects or because the subsidy raises their incomes. Another concern is that taxes are potentially regressive by disproportionately affecting lower-income households who spend a greater proportion of their household budget on food [9]. However, targeted taxes would affect households according to spending on individual items, not food spending overall. They could even have greater impacts on the health status of lower-income groups if their diets were improved more than those of higher-income groups, because of greater price sensitivity [10].

In New Zealand, significant ethnic and socioeconomic disparities exist for nutrition-related causes of death [11]–[13]. Information on the effects of food prices on consumer demand and differences across income levels and ethnic groups is therefore essential to estimate the effects of fiscal food policies on population health and inequalities. There is limited international evidence on the sensitivity of low-income populations to price changes [14], and a recent review highlighted the absence of data on responsiveness to food prices by culture or ethnicity [15]. A priori, we would expect that food demand elasticity would be greater amongst lower-income groups [16], consistent with the evidence for tobacco [17].

We aimed to estimate the effects of price changes on consumer demand for major commonly consumed food groups. Our goal was to provide a comprehensive summary of food demand and consumption behaviour, with particular attention to differences in price effects across income levels and ethnic groups.

## Methods

### Data sources

We used the following datasets to estimate own-price and cross-price elasticities for major food groups commonly consumed in New Zealand: Household Economic Surveys (HES) 2006/07 and 2009/10, and Food Price Index (FPI) data from 2007 and 2010.

The HES is conducted triennially and the target population is the usually resident population aged 15 years and over living in private dwellings. Data are collected using a household demographic questionnaire and an expenditure questionnaire that records larger purchases and regular payments made by a household over the previous 12-month period. Each eligible person in participating households is also asked to complete an expenditure diary recording all daily spending over a period of two weeks. Each HES is carried out over a full 12-month period. The 2006/07 HES included 2,902 households, while the 2009/10 HES included 3,126 households. Data from the two surveys were aggregated and used to develop PE values for New Zealand. Together these data included recorded household expenditures on 2,182 products, including 552 food items.

Whilst HES reports household expenditure on food it does not record the price of purchased foods which is an essential variable for demand analysis. We therefore derived food price information from the FPI. The FPI measures the change in food prices faced by households across New Zealand over time. Prices for a basket of 176 representative food items are collected monthly from 15 centres. The reference population for the FPI mirrors the target population for HES. Food pricing data from the 15 centres are aggregated to provide average monthly prices for six regions: Auckland, Canterbury, Wellington, North of the North Island, Rest of the South Island, and Rest of the North Island. The regional and monthly data provided the price variation required for the econometric analysis.

### Data matching

Overall HES recorded 552 food items whereas FPI provided price information for only 176 food items. For the remaining 376 foods in HES, we assumed their price to be the average weighted price of their counterparts in the same ‘within-class’ level.

FPI food categories could be readily matched with HES at food category and within-class level. For example, the HES food category ‘Fruit and Vegetables’ had a class level of ‘Fruit’ and within-class levels of ‘Citrus Fruit’, ‘Bananas’, and ‘Apples and Pears’. Matching of FPI data to HES data was undertaken as follows: (1) HES expenditure data were aggregated to the within-class level; (2) for products in both FPI and HES matching was undertaken using the common ‘within class’ product code across months and regions; and (3) for HES products not in the FPI, their prices were assumed to be the average prices of their counterparts within the same food class by regions and months. Prices were weighted by average quantity purchased (g or mL). Quantities were calculated using price per kg/L and total expenditure for the corresponding food group.

The aim was to develop own-PEs and cross-PEs for 24 food groups. The 24 food groups were chosen based on the following key parameters: 1) available FPI food group data; 2) a range of promising fiscal regimens (selected food taxes and subsidy policies) selected for exploration in subsequent analyses; and 3) identification of food groups that were major contributors to the nutrients/foods targeted by the selected fiscal policies. Expenditure and consumption of within-class food products were aggregated to the 24 food groups’ level for each household. The prices for each food group were calculated as the ratio between expenditure and quantity, which was essentially the quantity weighted average FPI price of the within-class level products. This is a common approach in demand analysis literature [18], [19]. Price was calculated as unit value.

As data were used from both 2006/07 and 2009/10 HES surveys, deflators were applied to food prices, food expenditure, and household income. For food prices and expenditures, we used Statistics New Zealand’s monthly aggregate food price index as a deflator. Prices and food expenditures were deflated to their value in June 2006. Household incomes were deflated using Statistics New Zealand’s Quarterly Consumer Price Index (CPI). Household incomes were deflated to their value in June Quarter 2006.

### Estimation of food price elasticities

To estimate food price elasticities, we used the linearized version of the Almost Ideal Demand System (LA/AIDS) methodology [20], the mainstay of household demand systems estimation of various expenditure items [21]. As is common in the literature, the basic LA/AIDS model was extended by including demographic variables (household type and size dummies; household ethnicity dummies based on ethnicity of household head; region dummies; month dummies; and income. Since the HES expenditure diary only covers two weeks, expenditure on some food items, such as rice and flour, may be zero for some households during this period. This would lead to inconsistent estimation if the censored nature of the data were not taken into account. Censored data (zero expenditure) was therefore dealt with in a two-stage analysis [22] by integrating the Heckman selection procedure into LA/AIDS model [23]. The first stage involved using a probit model to estimate individual households’ decision on whether to buy a food product or not; an inverse Mills’ ratio was then calculated for each household and product. At the second stage, the inverse Mills’ ratios were included as independent variables in the demand system estimation, and the calculation of price elasticities has also taken this into account. Robust standard errors for the coefficients were obtained using 400 bootstraps.

Household ethnicity was classified according to the ethnicity of the household head, defined as the person who completed the household demographic questionnaire. Households were classified by income based on Statistics New Zealand’s decimal classification. Quintile 1 in our analyses corresponds to Statistics New Zealand’s decimal 1 and 2 (lowest) categories, quintile 2 to decimal categories 3 and 4, and so forth. Food price elasticities were computed individually for each ethnic and income group. The dataset was disaggregated into ethnic and income subsets, and the demand system was estimated separately for each subset.

Income and ethnic differences in own-PE were quantified as follows. *Income*: For each food group, we ran an inverse-variance weighted ordinary least squares regression of the five quintile own-PEs (dependent variable) by the income quintiles’ relative position on a continuous scale (0 for highest income quintile, 0.25 for second quintile,…, and 1.0 for lowest income quintile). Accordingly, the regression coefficient gave the estimated difference in own-PE between the middle of the lowest income quintile and the middle of the highest income quintile. Second, we calculated ratios using regression predictions for the lowest to highest income quintile. Third, we calculated average absolute differences in own-PE between the lowest and highest income quintiles across all food groups, and the 95% confidence intervals for this average assuming (by necessity) no covariance in the beta coefficients from the separate models. Fourth, we calculated the geometric mean of the ratios across food groups. *Ethnicity*: For the Māori: non-Māori comparison, we calculated the equivalent of the income measures, but without the need to to use regression methods.

## Results

A total of 6,028 New Zealand households participated in the 2006/07 and 2009/10 HES surveys (**Table 1**). Twenty five per cent were located in Auckland, the largest city, and the remainder were approximately equally distributed across the country. Ten percent of households were classified as Māori (indigenous New Zealanders) based on the ethnicity of the person who completed the household demographic questionnaire; 4% were Pacific; and the remaining 86% were of European, Asian or other ethnicities. Recorded (non-equivalised) annual household incomes were split into quintiles and ranged from an average of $16,373 (Quintile 1, 2006/07) to $180,259 (Quintile 5, 2009/10). Most (59%) lived in one- or two-person households; with only 9% in households of five persons or more.

**Table 1:** Demographic Characteristics of Households Participating in 2006/07 and 2009/10 New Zealand Household Economic Surveys.

	N	Proportion
**Households**		
2006/07	2,902	48.1%
2009/10	3,126	52.9%
Total population	6,028	100%
**Households by region**		
North-North Island	867	14%
Auckland	1,477	25%
Middle North Island	713	12%
Wellington	938	16%
Canterbury	1,067	18%
Remainder of South Island	966	16%
**Households by ethnicity**		
Māori	578	10%
Pacific	216	4%
European/Asian/Other	5,234	86%
**Households by composition**		
Couple only	1,722	29%
Couple with child/children	1,726	29%
One parent with children	571	9%
One person household	1,302	22%
Others	707	12%
**Households by size**		
1 person	1,302	22%
2 person	2,231	37%
3 person	984	16%
4 person	945	16%
5 person or more	566	9%

**Figure 1:**
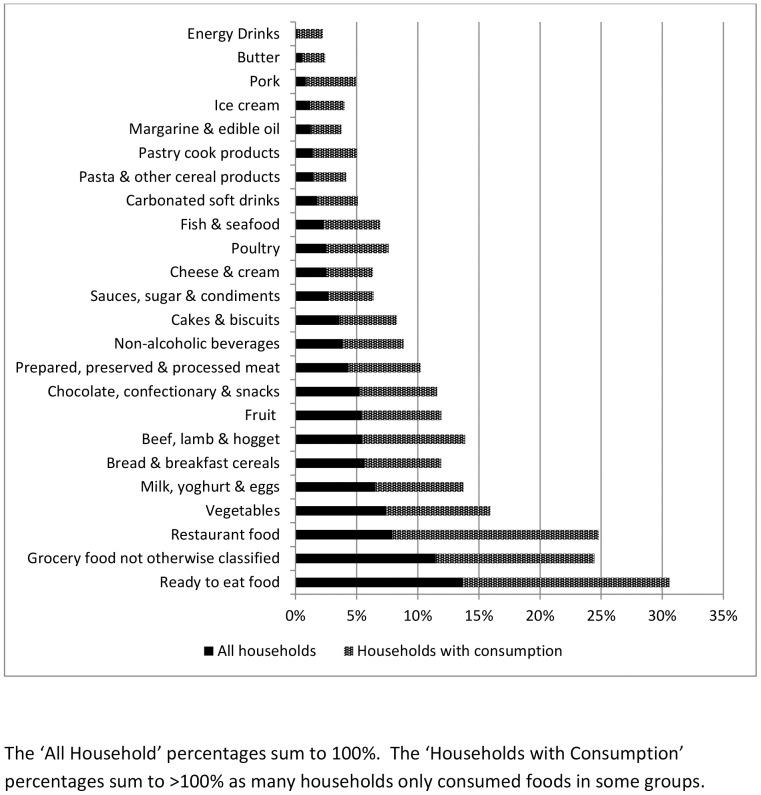
New Zealand Household Food Expenditure by Food Group, 2006 −**2010.**

In 2006/07 average annual household food expenditure was $7,630 (11% of income) and in 2009/10 it was $9,220 (12% of income). Food expenditure as a proportion of household income varied more than three-fold across income levels, comprising only 8% of income for households in the top quintile of income (5) in 2009/10 compared with 26% for those in the lowest quintile (1; **Table 2**). The top five food groups for expenditure were ready to eat (takeaway) foods (14% of expenditure share); grocery foods not otherwise classified (11.5%); restaurant food (8%); vegetables (7%); and milk, yoghurt and eggs (6.5%) (**Figure 1**).

**Table 2:** New Zealand Household Food Expenditure across Income Levels, 2006/07 and 2009/10.

	2006/07			2009/10		
	Average income	Average food expenditure	% income on food	Average income	Average food expenditure	% income on food
**Household income** *						
Quintile 1	$16,373	$3,823	23%	$18,921	$4,885	26%
Quintile 2	$34,132	$5,531	16%	$39,215	$6,660	17%
Quintile 3	$56,023	$7,035	13%	$63,200	$8,939	14%
Quintile 4	$81,362	$9,331	11%	$94,800	$11,151	12%
Quintile 5	$153,002	$12,423	8%	$180,259	$14,509	8%
Total population	$68,288	$7,630	11%	$78,405	$9,220	12%

* Non-equivalised.

**Table 3** reports the average food prices (by food group) paid by New Zealand households during each HES survey period and pooled across the survey periods. Little variation was evident in mean prices between survey years (on average 2009/10 inflation-adjusted prices were about 8% lower than in 2006/07). The variability (standard deviation) in food class price represents variation within each food group, and across survey periods, and six aggregated regions (see footnotes to Table 3). There was good variability in prices for econometric modelling of PE. However, there was also high variability within survey years, particularly for sauces, sugar and condiments; chocolate, confectionary and snacks (2010 only); ice cream; and non-alcoholic beverages. Possible reasons for this variability include store-to-store and region-to-region price variation, variation over the 12-month survey period, and product category aggregation (for example, the sauces, sugar and condiments class covers a range of diverse products with dissimilar prices).

**Table 3:** Food Prices (mean and standard deviation†) Paid by New Zealand Households in 2006/07 and 2009/10.

		2006/07			2008/09			2006/07 & 2008/09	
	Food Price ($/Kg)								
	N*	Mean	(SD)	N*	Mean	(SD)	N*	Mean	(SD)
Fruit	2416	3.32	(0.99)	2638	3.05	(0.82)	5056	3.18	(0.92)
Vegetables	2515	3.10	(1.13)	2787	2.96	(1.08)	5304	3.03	(1.11)
Beef, lamb & hogget	1886	11.92	(2.60)	2037	12.02	(2.43)	3925	11.97	(2.51)
Poultry	1383	6.64	(1.19)	1589	6.85	(1.03)	2974	6.75	(1.11)
Pork	607	12.11	(1.08)	653	12.42	(1.37)	1262	12.27	(1.25)
Prepared, preserved & processed meat	2130	12.74	(4.16)	2334	11.94	(3.63)	4466	12.32	(3.92)
Fish & seafood	1427	12.82	(3.74)	1613	13.05	(3.97)	3042	12.94	(3.86)
Bread & breakfast cereals	2547	3.71	(0.77)	2820	3.92	(0.62)	5369	3.82	(0.70)
Cakes & biscuits	2216	9.91	(1.01)	2409	10.10	(1.04)	4627	10.01	(1.03)
Pastry cook products	1295	6.21	(1.47)	1085	6.28	(1.31)	2382	6.24	(1.39)
Pasta & other cereal products	1523	3.66	(2.01)	1773	3.46	(1.81)	3298	3.56	(1.91)
Milk, yoghurt & eggs	2601	2.26	(1.04)	2873	2.45	(1.30)	5476	2.36	(1.19)
Cheese & cream	1869	8.60	(4.02)	2141	9.01	(4.25)	4012	8.81	(4.15)
Butter	772	3.96	(0.16)	970	5.37	(0.92)	1742	4.69	(0.98)
Margarine & edible oil	1391	6.20	(2.39)	1604	6.03	(2.00)	2997	6.11	(2.20)
Sauces, sugar & condiments	2073	9.07	(16.29)	2350	8.88	(16.14)	4425	8.97	(16.21)
Chocolate, confectionary & snacks	2329	17.22	(5.71)	2579	20.48	(17.34)	4910	18.91	(13.20)
Ice cream	1262	18.13	(12.95)	1392	18.01	(12.45)	2655	18.07	(12.69)
Other grocery food	2559	7.23	(1.61)	2775	7.60	(1.93)	5336	7.42	(1.79)
Non-alcoholic beverages	2209	8.02	(9.72)	2471	9.17	(9.88)	4682	8.62	(9.82)
Carbonated soft drinks	1532	2.26	(0.12)	1738	2.89	(0.14)	3270	2.59	(0.34)
Energy drinks	136	6.67	(0.29)	267	5.93	(0.31)	403	6.28	(0.48)
Restaurant food	1400	206.52	(48.02)	1416	169.14	(51.32)	2816	187.14	(53.15)
Ready to eat food	2333	15.16	(4.67)	2555	15.67	(4.82)	4890	15.42	(4.76)

† The mean and standard deviation was across the 12 (24) months in each (both) year(s), and the six aggregated regions (Auckland, Canterbury, Wellington, North of North Island, rest of North Island, rest of South Island).

* Number of households with consumption.

### Own price elasticity (PE) estimates

**Figure 2:**
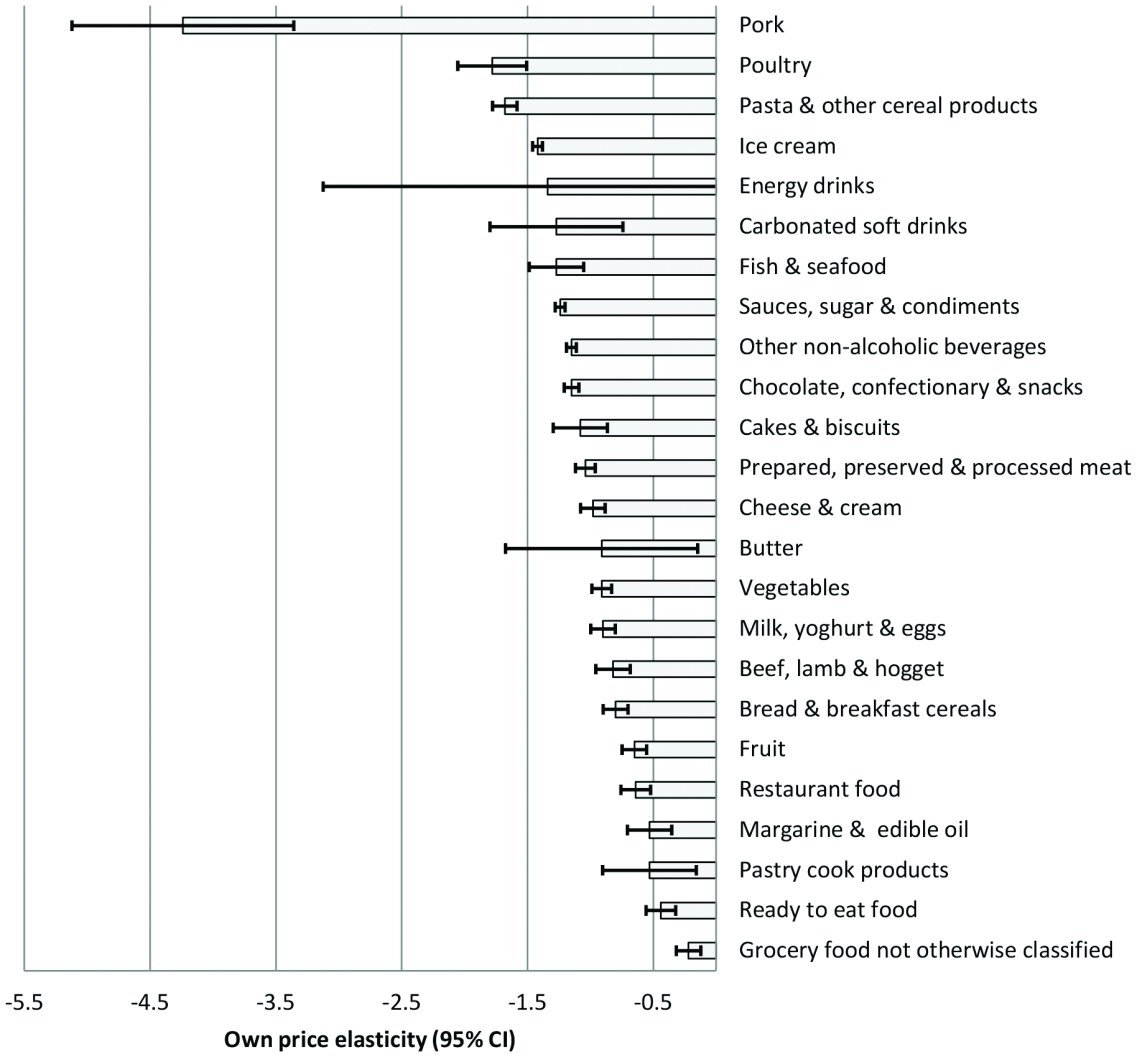
New Zealand Own-Price Food Elasticity Values by Food Group, 2006–2010.

Food price elasticities were estimated individually for 2006/07 and 2009/10 by running the AIDS model separately for each survey period. Data from both surveys were also aggregated to calculate combined estimates. PE estimates were largely consistent between survey years; however large differences were evident for a few categories, namely pastry cook products, butter, other grocery food, and energy drinks. Overall mean own-PE estimates (weighted average of both survey estimates) and their 95% confidence intervals (95% CI) are presented in **Figure 2**. Aside from pork (PE −4.24) and ‘grocery food not otherwise classified’ (PE −0.22), own-PE values spanned a range from −0.44 (ready to eat food; inelastic) to −1.78 (poultry; elastic). The average standard error (SE) across all own-PEs was 0.14. Nine food groups had own-PEs greater than −1 (i.e. closer to 0) with 95% CIs excluding −1 (i.e. consistent with customary characterisation of the demand response to food prices as inelastic). Twelve groups had own-PEs less than −1 (i.e. elastic), of which eight had 95% CIs excluding −1.

A number of PE values should be treated with caution, specifically pork (mean PE −4.24, SE 0.45); butter (mean −0.91, SE 0.39); energy drinks (mean −1.34, SE 0.91), and carbonated soft drinks (mean −1.27, SE 0.27). These categories had relatively small price variability and large standard errors. Finally, ‘grocery food not otherwise classified’ is a heterogeneous and residual food category, comprising spreads and dips, soup, prepared meals, canned meals, prepared desserts and baby food.

### PE estimates by income level

The effects of income level on demand elasticity are presented in **Table 4**. Across most food groups demand was more elastic among low-income households, indicating these households are more sensitive to price changes. Weighted OLS regressions of the quintile PE estimates are presented in the penultimate column of Table 4. Excluding the outlier of ‘energy drinks’, nine of the 23 food groups had statistically significant stronger own-PEs for the lowest versus highest income quintile (i.e. 95% CI excluded the null of zero difference by income), with the average regression-based difference being −0.30 (95% CI −0.62 to 0.02).

**Table 4:** New Zealand Own-Price Food Elasticity Values by Income Quintile (lowest to highest), 2006−2010.

Food Group	Own-PE estimate (standard error)						OLS regression‡ estimated PE differences Quintile 1 vs 5	
	Quintile 1	Quintile 2	Quintile 3	Quintile 4	Quintile 5	Total population	Absolute (95% CI)*	Ratio^£^
Fruit	−0.78 (0.17)	−0.70 (0.10)	−0.51 (0.12)	−0.65 (0.15)	−0.66 (0.11)	−0.65 (0.05)	−0.08 (−0.34 to 0.17)	1.13
Vegetables	−1.09 (0.10)	−1.04 (0.08)	−0.80 (0.08)	−0.82 (0.06)	−0.64 (0.08)	−0.91 (0.04)	−**0.44 (**−**0.62 to** −**0.26)**	1.64
Beef, lamb & hogget	−1.26 (0.23)	−0.77 (0.19)	−0.61 (0.14)	−0.86 (0.14)	−0.75 (0.15)	−0.82 (0.07)	−0.23 (−0.82 to 0.36)	1.33
Poultry	−2.47 (0.52)	−1.75 (0.39)	−1.69 (0.26)	−1.68 (0.26)	−1.33 (0.20)	−1.78 (0.14)	−**0.82 (**−**1.27 to** −**0.37)**	1.60
Pork	−3.26 (1.19)	−5.64 (1.39)	−2.51 (0.82)	−3.35 (0.75)	−5.29 (1.24)	−4.24 (0.45)	0.65 (−3.33 to 4.63)	0.86
Prepared, preserv. & process. meat	−1.01 (0.13)	−1.21 (0.10)	−1.01 (0.07)	−1.01 (0.08)	−0.87 (0.10)	−1.04 (0.04)	−0.23 (−0.50 to 0.04)	1.25
Fish & seafood	−1.71 (0.35)	−1.19 (0.35)	−1.21 (0.19)	−1.42 (0.21)	−1.42 (0.16)	−1.27 (0.11)	0.02 (−0.48 to 0.53)	0.98
Bread & breakfast cereals	−1.15 (0.15)	−0.71 (0.10)	−0.70 (0.09)	−0.69 (0.10)	−0.48 (0.14)	−0.80 (0.05)	−**0.45 (**−**0.83 to** −**0.07)**	1.77
Cakes & biscuits	−1.30 (0.29)	−1.16 (0.24)	−1.09 (0.23)	−0.69 (0.20)	−1.31 (0.24)	−1.08 (0.11)	−0.21 (−1.02 to 0.60)	1.21
Pastry cook products	−2.17 (0.94)	−1.19 (0.48)	−0.95 (0.33)	−0.08 (0.28)	−0.75 (0.33)	−0.53 (0.19)	−1.10 (−2.78 to 0.58)	*undef*
Pasta & other cereal products	−2.23 (0.18)	−1.70 (0.12)	−1.59 (0.10)	−1.67 (0.11)	−1.35 (0.08)	−1.68 (0.05)	−**0.61 (**−**1.01 to** −**0.22)**	1.45
Milk, yoghurt & eggs	−1.05 (0.14)	−1.00 (0.11)	−0.93 (0.07)	−0.62 (0.16)	−0.98 (0.08)	−0.90 (0.05)	−0.08 (−0.41 to 0.26)	1.09
Cheese & cream	−1.01 (0.15)	−1.12 (0.10)	−0.98 (0.08)	−1.03 (0.10)	−0.69 (0.08)	−0.98 (0.05)	−**0.40 (**−**0.74 to** −**0.07)**	1.52
Butter	−2.44 (1.48)	−2.16 (0.68)	−0.27 (0.68)	−1.08 (0.72)	−2.07 (0.71)	−0.91 (0.39)	−0.21 (−3.17 to 2.75)	1.26
Margarine & edible oil	−0.54 (0.30)	−0.78 (0.17)	−0.73 (0.16)	−0.46 (0.19)	−0.40 (0.14)	−0.53 (0.09)	−0.40 (−0.74 to −0.06)	2.20
Sauces, sugar & condiments	−1.37 (0.06)	−1.30 (0.06)	−1.20 (0.04)	−1.17 (0.03)	−1.13 (0.03)	−1.24 (0.02)	−**0.22 (**−**0.29 to** −**0.16)**	1.20
Chocolate, confectionary & snacks	−1.30 (0.09)	−1.18 (0.10)	−1.14 (0.07)	−1.13 (0.07)	−1.17 (0.06)	−1.15 (0.03)	−0.10 (−0.23 to 0.04)	1.09
Ice cream	−1.75 (0.07)	−1.58 (0.06)	−1.41 (0.05)	−1.21 (0.05)	−1.24 (0.04)	−1.42 (0.02)	−**0.51 (**−**0.71 to** −**0.30)**	1.43
Other grocery foods	−0.55 (0.16)	−0.10 (0.12)	−0.09 (0.11)	−0.18 (0.11)	−0.24 (0.12)	−0.22 (0.05)	−0.11 (−0.58 to 0.36)	1.74
Non-alcoholic beverages	−1.08 (0.06)	−1.20 (0.03)	−1.24 (0.03)	−1.17 (0.03)	−1.08 (0.03)	−1.15 (0.02)	−0.10 (−0.29 to 0.09)	1.09
Carbonated soft drinks	−2.20 (1.16)	−3.47 (0.99)	−0.14 (0.43)	−2.95 (0.52)	−1.26 (0.44)	−1.27 (0.27)	0.07 (−4.71 to 4.84)	0.95
Energy drinks	−5.05 (8.59)	−2.96 (3.22)	−2.28 (1.72)	−1.12 (1.51)	−0.78 (2.02)	−1.34 (0.91)	−**3.35 (**−**4.49 to** −**2.21)**	*undef*
Restaurant food	−0.73 (0.35)	−1.09 (0.20)	−0.39 (0.15)	−0.86 (0.10)	−0.68 (0.08)	−0.64 (0.06)	−0.11 (−0.86 to 0.64)	1.20
Ready to eat food	−2.09 (0.33)	−0.50 (0.17)	−0.46 (0.10)	−0.09 (0.11)	−0.01 (0.11)	−0.44 (0.06)	−**1.15 (**−**2.04 to** −**0.25)**	6.87
**Average across foods**		*Including All Products* *Excluding Energy Drinks*					−**0.42 (**−**0.73 to** −**0.11)†**	*undef*
							−0.30 (−0.62 to 0.02)†	*undef*
		*Excluding Energy Drinks and Pastry Cook products*					−0.26 (−0.59 to 0.07)†	1.40

* Bold if 95% CI excludes the null. ** Geometric mean given ratios, and excluding ‘undefined’ estimates. † Calculated assuming no covariance. ‡ Ordinary least squares regression using the inverse of the variance of each PE as the weight, and the midpoints of the quintiles coded a 0, 0.25, 0.5, 0.75 and 1.0 such that the beta coefficient gives the change from the middle. ^£^ Calculated as (Total own-PE+(0.5×absolute))/(Total own-PE −(0.5×absolute)), where: total own-PE  =  the own-PE before stratifying by income; absolute  =  the OLS regression estimated absolute difference in own-PE between the first and fifth quintiles of income. For example, for fruit the ratio  =  (−0.65 + (0.5×−0.08))/(−0.65 − (0.5×−0.08))  =  1.13.

*Undef*  =  undefined (due to OLS regression estimate predicting a positive own-PE for the high income quintile).

The final column of Table 4 shows the regression-based ratio of own-PE for the lowest compared to the highest income quintile. Averaged across food groups (geometric mean; excluding the undefined ratios for ‘Energy Drinks’ and ‘Pastry Cook Products’), the own-PE was 40% stronger among low- compared to high-income quintiles.

### PE estimates by ethnic group

The variations by ethnicity in demand elasticity are presented in **Table 5**. Differences in estimated PEs were evident between Māori, non-Māori, and non-Māori non-Pacific (predominantly European) households, although estimates were often unstable due to small numbers. Nevertheless, there was a pattern across food groups of greater demand elasticity among Māori households. For example, six of the 24 absolute differences in own-PEs between Māori and non-Māori were statistically significantly stronger among Māori (i.e. 95% CI excluding the null). A separate analysis where interaction terms between prices and ethnic groups were included in the same regression and a t-test was conducted to compare the coefficients of different interaction terms produced very similar results (data not shown). Excluding the outlier ‘Energy Drinks’, the average difference across the 23 food groups was −0.26 (95% CI −0.52 to −0.00). The final column of Table 5 shows the ratio of the Māori to non-Māori own-PE. Averaged across food groups the own-PE was 25% stronger amongst Māori compared to non-Māori (15% excluding ‘Energy Drinks’).

**Table 5:** New Zealand Own-Price Food Elasticity Values by Ethnic Group, 2006−2010.

	Own PE						Difference Māori: non-Māori	
	Māori		Non-Māori		Non-Māori Non-Pacific			
Food Group	Mean	SE	Mean	SE	Mean	SE	Absolute *	Ratio
Fruit	−0.69	0.38	−0.64	0.05	−0.61	0.05	−0.05 (−0.80 to 0.70)	1.08
Vegetables	−1.33	0.15	−0.87	0.04	−0.87	0.04	−**0.46 (**−**0.76 to** −**0.16)**	1.53
Beef, lamb & hogget	−0.69	0.28	−0.83	0.07	−0.80	0.08	0.14 (−0.43 to 0.71)	0.83
Poultry	−3.13	0.58	−1.64	0.13	−1.58	0.13	−**1.49 (**−**2.66 to** −**0.32)**	1.91
Pork	−4.37	1.44	−4.10	0.48	−4.19	0.50	−0.27 (−3.25 to 2.71)	1.07
Prepared, preserved & processed meat	−0.91	0.16	−1.04	0.04	−1.02	0.04	0.13 (−0.19 to 0.45)	0.88
Fish & seafood	−1.69	0.32	−1.20	0.11	−1.14	0.10	−0.49 (−1.15 to 0.17)	1.41
Bread & breakfast cereals	−1.16	0.20	−0.74	0.05	−0.74	0.05	−**0.42 (**−**0.82 to** −**0.02)**	1.57
Cakes & biscuits	−0.93	0.46	−1.09	0.11	−1.15	0.11	0.16 (−0.77 to 1.09)	0.85
Pastry cook products	−2.08	0.78	−0.31	0.18	−0.27	0.19	−**1.77 (**−**3.34 to** −**0.20)**	6.71
Pasta & other cereal products	−1.62	0.21	−1.68	0.05	−1.69	0.05	0.06 (−0.36 to 0.48)	0.96
Milk, yoghurt & eggs	−0.68	0.17	−0.92	0.05	−0.91	0.06	0.24 (−0.11 to 0.59)	0.74
Cheese & cream	−1.28	0.21	−0.95	0.06	−0.95	0.05	−0.33 (−0.76 to 0.10)	1.35
Butter	−1.68	1.33	−0.95	0.44	−0.91	0.43	−0.73 (−3.48 to 2.02)	1.77
Margarine & edible oil	−1.65	0.26	−0.45	0.10	−0.45	0.10	−**1.20 (**−**1.75 to** −**0.65)**	3.67
Sauces, sugar & condiments	−1.34	0.08	−1.23	0.02	−1.21	0.02	−0.11 (−0.27 to 0.05)	1.09
Chocolate, confectionary & snacks	−1.03	0.19	−1.16	0.03	−1.15	0.03	0.13 (−0.25 to 0.51)	0.89
Ice cream	−1.25	0.09	−1.43	0.02	−1.42	0.02	0.18 (0.00 to 0.36)	0.87
Other grocery food	−0.11	0.20	−0.25	0.05	−0.26	0.06	0.14 (−0.26 to 0.54)	0.44
Non−alcoholic beverages	−1.31	0.11	−1.13	0.02	−1.13	0.02	−0.18 (−0.40 to 0.04)	1.16
Carbonated soft drink	−1.11	0.83	−1.38	0.30	−1.34	0.30	0.27 (−1.46 to 2.00)	0.80
Energy drinks	−7.92	5.08	−0.93	1.01	−1.04	0.98	−6.99 (−17.14 to 3.16)	8.52
Restaurant food	−0.09	0.31	−0.68	0.06	−0.66	0.06	0.59 (−0.03 to 1.21)	0.13
Ready to eat food	−0.88	0.15	−0.32	0.07	−0.24	0.06	−**0.56 (**−**0.88 to** −**0.24)**	2.75
**Average across foods**	*Including Energy Drinks*						−**0.54 (**−**0.85 to** −**0.23) †**	1.25**
	*Excluding Energy Drinks*						−**0.26 (**−**0.52 to 0.00) †**	1.15**

* Bold if 95% CI excludes the null ** Geometric mean given ratios. † Calculated assuming no covariance.

### Cross-PE estimates

Estimation of cross-PEs enables assessment of likely between-category shifts in food purchasing in response to price changes that could support or undermine the health objective of a food tax or subsidy. Larger cross-PEs indicate that non-targeted foods are suitable complements or substitutes for targeted (taxed/subsidised) foods, and thus there is a higher likelihood of compensatory purchasing as prices shift. Total population cross-PE estimates and standard errors are presented in **Table 6**.

**Table 6:** New Zealand Cross-Price Food Elasticity Values (Means and Standard Errors (SE)), 2006−2010*.

	Fruit	Vegetables	Beef, Lamb & hogget	Poultry	Pork	Prepared, preserved & processed meat	Fish & seafood	Bread & breakfast cereals	Cakes & biscuits	Pastry cook products	Pasta & other cereal products	Milk, yoghurt & eggs	Cheese & cream	Butter	Margarine & edible oil	Sauces, sugar & condiments	Chocolate, confectionary & snacks	Ice cream	Other grocery food	Non-alcoholic beverages	Carbonated soft drink	Energy drinks	Restaurant food	Ready to eat food
Fruit	−**0.65 (0.05)**	0.03 (0.03)	−**0.11 (0.04)**	0.03 (0.03)	**0.05 (0.02)**	−**0.07 (0.03)**	−0.00 (0.02)	0.16 (0.03)	−**0.32 (0.04)**	**0.08 (0.02)**	−0.05 (0.01)	**0.03 (0.03)**	**0.08 (0.02)**	0.11 (0.01)	−0.07 (0.02)	−0.01 (0.03)	−**0.07 (0.03)**	−0.01 (0.01)	−**0.33 (0.04)**	−0.01 (0.01)	−0.12 (0.03)	0.39 (0.02)	**0.01 (0.03)**	−**0.22 (0.04)**
Vegetables	0.01 (0.02)	−**0.91 (0.04)**	−**0.09 (0.03)**	**0.01 (0.02)**	−0.01 (0.01)	−**0.03 (0.02)**	0.03 (0.02)	**0.01 (0.02)**	−**0.24 (0.02)**	−0.02 (0.01)	**0.02 (0.01)**	−**0.01 (0.02)**	−0.02 (0.01)	0.02 (0.01)	−0.04 (0.01)	**0.04 (0.01)**	−0.06 (0.02)	−0.02 (0.01)	−**0.30 (0.04)**	0.02 (0.01)	−**0.02 (0.02)**	0.11 (0.01)	**0.10 (0.03)**	−0.10 (0.03)
Beef, lamb & hogget	−**0.18 (0.04)**	−**0.18 (0.04)**	−**0.82 (0.07)**	0.13 (0.04)	−**0.01 (0.02)**	−0.04 (0.04)	−0.08 (0.03)	−**0.20 (0.04)**	−**0.18 (0.04)**	−0.07 (0.03)	−0.03 (0.02)	−0.05 (0.04)	−0.07 (0.03)	0.19 (0.02)	−0.05 (0.02)	−**0.06 (0.02)**	−0.03 (0.04)	**0.00 (0.01)**	−**0.33 (0.06)**	0.06 (0.02)	−0.23 (0.04)	0.12 (0.01)	**0.14 (0.04)**	0.08 (0.06)
Poultry	−0.12 (0.07)	−0.14 (0.06)	−**0.01 (0.08)**	−**1.78 (0.14)**	0.01 (0.06)	**0.07 (0.05)**	0.00 (0.05)	0.01 (0.09)	**0.11 (0.09)**	**0.12 (0.06)**	0.01 (0.03)	**0.07 (0.06)**	−0.01 (0.04)	−0.13 (0.05)	0.08 (0.04)	**0.02 (0.03)**	0.01 (0.05)	**0.00 (0.02)**	−0.18 (0.09)	**0.07 (0.02)**	**0.35 (0.09)**	−0.07 (0.03)	−**0.02 (0.06)**	0.10 (0.07)
Pork	0.23 (0.11)	0.07 (0.10)	0.30 (0.13)	0.09 (0.19)	−**4.24 (0.45)**	−**0.02 (0.08)**	−0.03 (0.13)	0.09 (0.13)	**0.97 (0.26)** ‡	0.37 (0.11)	−0.01 (0.04)	−**0.05 (0.09)**	0.08 (0.06)	0.18 (0.16)	0.20 (0.09)	0.06 (0.04)	**0.25 (0.11)**	0.08 (0.02)	0.04 (0.10)	−**0.01 (0.03)**	**0.74 (0.28)**	**0.28 (0.11)**	−0.17 (0.08)	0.24 (0.11)
Prepared, preserved & processed meat	−**0.13 (0.03)**	−0.11 (0.04)	−0.06 (0.05)	0.20 (0.03)	0.09 (0.02)	−**1.04 (0.04)**	0.01 (0.02)	−**0.10 (0.03)**	−0.11 (0.03)	−0.02 (0.02)	0.08 (0.02)	−**0.08 (0.03)**	**0.02 (0.02)**	0.15 (0.01)	−0.12 (0.02)	−0.08 (0.01)	−0.18 (0.03)	−0.05 (0.01)	−0.24 (0.04)	0.00 (0.01)	−0.14 (0.03)	0.01 (0.01)	**0.18 (0.03)**	−**0.16 (0.04)**
Fish & seafood	0.03 (0.06)	0.03 (0.06)	−0.02 (0.07)	0.03 (0.06)	0.03 (0.05)	0.01 (0.05)	−**1.27 (0.11)**	0.03 (0.06)	0.08 (0.05)	−0.06 (0.04)	0.07 (0.03)	**0.05 (0.05)**	−0.00 (0.03)	0.01 (0.02)	0.02 (0.03)	0.05 (0.03)	0.06 (0.05)	0.00 (0.02)	−0.06 (0.09)	0.04 (0.02)	0.06 (0.05)	0.56 (0.11)	−**0.05 (0.07)**	−0.01 (0.07)
Bread & breakfast cereals	−**0.03 (0.03)**	−0.09 (0.02)	−**0.11 (0.04)**	−0.05 (0.04)	−0.04 (0.02)	−**0.08 (0.03)**	0.02 (0.02)	−**0.80 (0.05)**	−0.05 (0.04)	−0.10 (0.03)	0.00 (0.02)	−**0.20 (0.03)**	0.01 (0.02)	0.07 (0.02)	−**0.12 (0.02)**	−0.04 (0.01)	−**0.01 (0.03)**	−0.02 (0.01)	−**0.26 (0.03)**	0.00 (0.01)	−0.03 (0.04)	−0.15 (0.01)	**0.07 (0.03)**	−0.09 (0.03)
Cakes & biscuits	−**0.17 (0.05)**	−**0.24 (0.04)**	−**0.15 (0.06)**	0.19 (0.06)	**0.17 (0.06)**	−**0.03 (0.04)**	0.05 (0.03)	0.06 (0.06)	−**1.08 (0.11)**	−**0.07 (0.05)**	−0.02 (0.02)	−**0.03 (0.04)**	−**0.06 (0.03)**	0.07 (0.04)	−0.17 (0.03)	−0.04 (0.02)	−**0.10 (0.03)**	−**0.05 (0.01)**	−0.09 (0.05)	**0.00 (0.01)**	0.03 (0.08)	0.37 (0.02)	0.10 (0.04)	−0.13 (0.05)
Pastry cook products	**0.22 (0.09)**	0.10 (0.07)	−0.03 (0.11)	0.32 (0.11)	**0.04 (0.06)**	0.05 (0.07)	0.10 (0.07)	**0.15 (0.14)**	−0.52 (0.15)	−**0.53 (0.19)**	0.02 (0.04)	0.31 (0.09)	0.05 (0.06)	−0.66 (0.08)	0.33 (0.07)	0.04 (0.03)	0.23 (0.06)	**0.05 (0.03)**	−**0.09 (0.11)**	0.06 (0.03)	**0.95 (0.19)** ‡	−**1.14 (0.12)** ‡	**0.05 (0.08)**	**0.37 (0.09)**
Pasta & other cereal products	−**0.07 (0.06)**	0.16 (0.05)	−0.13 (0.08)	−0.08 (0.06)	−0.01 (0.03)	**0.09 (0.05)**	−0.00 (0.05)	0.02 (0.06)	0.04 (0.06)	0.08 (0.04)	−**1.67 (0.05)**	0.01 (0.05)	−0.00 (0.04)	−0.11 (0.02)	−0.03 (0.03)	−**0.02 (0.03)**	0.00 (0.05)	0.03 (0.02)	0.08 (0.08)	0.07 (0.02)	0.14 (0.06)	0.07 (0.03)	−0.11 (0.06)	0.15 (0.10)
Milk, yoghurt & eggs	−0.09 (0.03)	−0.11 (0.03)	−0.00 (0.03)	0.11 (0.02)	0.03 (0.01)	−**0.08 (0.02)**	0.05 (0.02)	−**0.13 (0.02)**	−0.14 (0.02)	−**0.02 (0.02)**	0.00 (0.01)	−**0.90 (0.05)**	−0.03 (0.01)	0.19 (0.11)	−0.03 (0.01)	−**0.02 (0.01)**	−**0.01 (0.02)**	−0.00 (0.01)	−0.25 (0.04)	**0.04 (0.01)**	−0.09 (0.02)	0.16 (0.01)	**0.05 (0.03)**	−0.01 (0.04)
Cheese & cream	**0.13 (0.05)**	0.03 (0.04)	−**0.10 (0.05)**	0.00 (0.04)	−0.01 (0.02)	0.12 (0.04)	0.08 (0.03)	0.10 (0.04)	−0.12 (0.05)	0.00 (0.03)	−0.01 (0.02)	0.01 (0.04)	−**0.98 (0.05)**	**0.21 (0.03)**	−0.09 (0.03)	−**0.08 (0.02)**	−**0.19 (0.04)**	−0.02 (0.02)	−**0.25 (0.06)**	**0.02 (0.02)**	0.06 (0.05)	0.04 (0.01)	**0.04 (0.05)**	−**0.31 (0.05)**
Butter	**0.05 (0.13)**	0.15 (0.10)	−**0.05 (0.14)**	**0.19 (0.19)**	0.41 (0.25)	**0.11 (0.10)**	−0.02 (0.08)	**0.23 (0.17)**	0.13 (0.27)	**0.01 (0.15)**	**0.01 (0.07)**	−**0.07 (0.11)**	**0.23 (0.10)**	−**0.91 (0.39)**	0.09 (0.09)	**0.09 (0.05)**	**0.25 (0.07)**	0.08 (0.02)	**0.10 (0.16)**	−0.03 (0.03)	−0.28 (0.42)	−0.04 (0.18)	−**0.10 (0.10)**	0.22 (0.14)
Margarine & edible oil	−**0.11 (0.08)**	−**0.00 (0.07)**	0.13 (0.10)	0.10 (0.09)	**0.09 (0.06)**	−**0.02 (0.06)**	0.07 (0.05)	−**0.22 (0.09)**	−**0.12 (0.09)**	**0.10 (0.07)**	0.00 (0.04)	**0.05 (0.06)**	−**0.01 (0.05)**	0.00 (0.05)	−**0.53 (0.09)**	−0.01 (0.03)	−**0.02 (0.05)**	0.04 (0.02)	−**0.13 (0.09)**	0.04 (0.02)	0.06 (0.09)	0.23 (0.04)	−0.09 (0.07)	−0.05 (0.08)
Sauces, sugar & condiments	0.06 (0.03)	**0.12 (0.03)**	−**0.08 (0.04)**	0.07 (0.03)	−0.00 (0.01)	−**0.03 (0.02)**	**0.03 (0.02)**	−**0.03 (0.02)**	−0.03 (0.02)	0.01 (0.02)	−0.05 (0.02)	0.07 (0.03)	−**0.09 (0.02)**	0.08 (0.01)	−0.02 (0.01)	−**1.24 (0.02)**	−**0.14 (0.02)**	−**0.02 (0.01)**	−0.16 (0.05)	**0.01 (0.01)**	0.00 (0.02)	−0.02 (0.00)	−0.04 (0.03)	−0.04 (0.04)
Chocolate, confectionary & snacks	**0.07 (0.03)**	**0.03 (0.03)**	−0.03 (0.04)	−0.00 (0.02)	**0.12 (0.02)**	−**0.01 (0.02)**	−0.03 (0.02)	0.19 (0.03)	**0.07 (0.02)**	**0.01 (0.01)**	0.01 (0.01)	0.06 (0.03)	−**0.06 (0.02)**	**0.11 (0.01)**	−0.03 (0.01)	−**0.06 (0.01)**	−**1.15 (0.03)**	−0.05 (0.04)	−0.05 (0.04)	−**0.07 (0.01)**	0.21 (0.03)	0.33 (0.01)	0.00 (0.03)	−0.05 (0.04)
Ice cream	0.01 (0.04)	0.04 (0.04)	0.20 (0.06)	−0.41 (0.06)	**0.05 (0.02)**	−**0.04 (0.04)**	0.01 (0.03)	0.01 (0.04)	−**0.23 (0.04)**	0.09 (0.03)	0.00 (0.02)	0.09 (0.05)	0.03 (0.02)	0.02 (0.01)	0.04 (0.02)	0.06 (0.02)	0.00 (0.04)	−**1.42 (0.03)**	**0.36 (0.12)**	0.10 (0.02)	−**0.02 (0.03)**	0.26 (0.03)	−0.18 (0.07)	0.18 (0.09)
Other grocery food	−**0.08 (0.02)**	−**0.06 (0.02)**	−**0.16 (0.03)**	**0.01 (0.02)**	0.00 (0.01)	−**0.03 (0.02)**	−0.03 (0.02)	−**0.04 (0.02)**	−0.01 (0.02)	0.08 (0.02)	0.02 (0.01)	−**0.04 (0.02)**	−**0.05 (0.03)**	0.03 (0.01)	−0.04 (0.01)	−**0.01 (0.01)**	0.02 (0.02)	**0.03 (0.01)**	−**0.22 (0.05)**	0.00 (0.01)	−0.05 (0.01)	0.12 (0.01)	−0.13 (0.04)	−**0.13 (0.04)**
Non−alcoholic beverages	−0.01 (0.02)	0.01 (0.02)	**0.05 (0.02)**	**0.06 (0.01)**	0.05 (0.01)	0.02 (0.02)	0.03 (0.01)	0.03 (0.02)	−0.05 (0.02)	−0.02 (0.01)	**0.01 (0.01)**	**0.09 (0.02)**	0.05 (0.01)	0.00 (0.00)	0.01 (0.01)	−0.03 (0.01)	−**0.08 (0.02)**	−**0.01 (0.01)**	−0.08 (0.05)	−**1.15 (0.02)**	**0.06 (0.03)**	−0.10 (0.02)	−**0.10 (0.04)**	−**0.29 (0.04)**
Carbonated soft drinks	−**0.15 (0.09)**	−0.30 (0.08)	**0.17 (0.11)**	**0.41 (0.12)**	**0.39 (0.14)**	**0.09 (0.06)**	−0.07 (0.05)	−0.07 (0.13)	0.07 (0.13)	−**0.39 (0.09)**	**0.06 (0.04)**	**0.01 (0.07)**	0.06 (0.06)	0.05 (0.13)	−0.06 (0.06)	0.03 (0.03)	**0.05 (0.08)**	0.06 (0.02)	**0.06 (0.09)**	−**0.07 (0.03)**	−**1.27 (0.27)**	−0.25 (0.11)	−**0.03 (0.08)**	−0.02 (0.09)
Energy drinks	**0.13 (0.22)**	−**0.11 (0.19)**	**0.00 (0.30)**	**0.54 (0.43)**	**2.70 (0.69)**	0.36 (0.17)	**0.15 (0.13)**	**0.29 (0.26)**	**0.05 (0.46)**	−**0.45 (0.35)**	−**0.07 (0.10)**	**0.43 (0.22)**	0.28 (0.14)	0.31 (0.71)	**0.38 (0.19)**	0.02 (0.07)	**0.04 (0.12)**	**0.04 (0.07)**	−**0.57 (0.22)**	**0.13 (0.06)**	−**1.04 (1.10)** ‡	−1.34 (1.10)	**0.15 (0.22)**	−0.12 (0.18)
Restaurant food	0.06 (0.02)	**0.16 (0.02)**	**0.12 (0.03)**	0.05 (0.02)	0.00 (0.01)	**0.10 (0.02)**	0.06 (0.02)	0.12 (0.02)	**0.04 (0.02)**	0.07 (0.01)	0.01 (0.01)	0.07 (0.03)	0.04 (0.02)	−0.05 (0.01)	0.00 (0.01)	−0.01 (0.01)	−0.03 (0.02)	**0.01 (0.01)**	−**0.06 (0.05)**	−**0.07 (0.02)**	−0.06 (0.02)	0.12 (0.02)	−**0.64 (0.06)**	**0.03 (0.05)**
Ready to eat food	−**0.22 (0.02)**	−**0.11 (0.02)**	0.13 (0.03)	0.07 (0.02)	**0.19 (0.02)**	−**0.11 (0.02)**	−0.00 (0.01)	−**0.25 (0.03)**	−**0.19 (0.02)**	**0.09 (0.02)**	0.09 (0.02)	0.10 (0.02)	−**0.13 (0.01)**	0.03 (0.01)	0.01 (0.01)	0.03 (0.01)	−0.03 (0.02)	**0.00 (0.01)**	−**0.25 (0.04)**	−**0.02 (0.01)**	0.19 (0.02)	0.32 (0.03)	−0.13 (0.03)	−**0.44 (0.06)**

* Text in bold if 95% CI excludes the null.

‡ Estimates are problematic due to high SE for –PE.

The average cross-PE value was 0.014 and 5th, 25th, median, 75th and 95th percentiles were −0.22, −0.05, 0.00, 0.07 and 0.27 respectively. Of the 552 cross-PEs, absolute values for 170 (31%) were greater than 0.10, 70 (13%) greater than 0.20, and 36 (6.5%) greater than 0.3. 293 (53%) cross-PEs had a 95% confidence interval excluding 0, and so can be considered “statistically significant” (shaded cells in Table 6). Taking into account magnitude of cross-PE values and their statistical and public health significance, cross-PEs of greatest potential importance include those between fruit and cakes/biscuits (−0.32 (SE 0.04)), fruit and ready-to-eat food (−0.22 (SE 0.04)), vegetables and cakes/biscuits (−0.24 (SE 0.02)), and cheese/cream and ready-to-eat food (−0.31 (SE 0.05)). Of note is that all these cross-PEs were less than zero, indicating that the products are complements i.e. as the price of one rises, purchases of both decrease. Conversely, if the price of one product decreases, purchases of both will increase.

## Discussion

Own-PE estimates (with two exceptions) for 24 food groups in New Zealand ranged from −0.44 to −1.78. Cross-PE estimates were small, with an average absolute value of 0.11. Differences were evident across income levels and ethnic groups. Averaged across 23 food groups, the own-PE was −0.30 stronger in the lowest compared to highest household income quintile, and −0.26 stronger amongst Māori compared to non-Māori.

Our PE estimates are generally higher than those from comparable countries. A review of 160 US-based studies between 1938 and 2007 reported food PEs ranging from 0.27 (95% CI 0.08, 0.45) to 0.81 (95% CI 0.56, 1.07), with food away from home, soft drinks, juice, and meats most responsive to price changes [15]. United Kingdom (UK) food PEs over the period 1988 to 2000 ranged from −0.17 (SE 0.15) to −0.94 (SE 0.10), and cereals and cereal products, fresh fish, and sugar and preserves were most responsive to price changes [24]. Pooled data from 114 countries described a narrow range of PEs for high-income countries from −0.14 (meat) to −0.36 (bread and cereals) [25]. Demand response to food price changes was larger in poorer countries however: comparable food PE ranges were −0.30 to −0.68 for middle-income countries, and −0.43 to −1.01 for low-income countries [25]. A recent meta-analysis of own-PE data from 162 countries also reported a similar pattern: PE values ranged from −0.36 to −0.77 in high-income countries compared with −0.54 to −0.95 in low-income countries [16].

Earlier New Zealand PE estimates for seven food groups based on 1996 expenditure and price data spanned a narrow range from −0.22 (bread and cereals) to −0.47 (fish) [25]. However, analysis of data from two Australian food expenditure surveys covering the period 1998 to 2004 reported own-PEs that ranged from −0.23 (milk) to −2.66 (rice), with values approximating or exceeding −1.0 for 10 of the 15 food categories examined [26].

There are a number of potential explanations for our higher NZ PE estimates. The first is that differences in estimates reflect the highly diverse methodologies used to derive PEs for different countries. Key differences in years of data collection; population sample sizes; numbers and types of food categories analysed; data quality and availability; and types of demand models used could explain much of the variability observed.

A related reason may be the comparatively small population sample and short measurement period from which the New Zealand estimates were derived. Our PE estimates were derived from two national household expenditure surveys (6,028 households) over the period 2006 to 2010. Whilst these were the best datasets available nationally for this purpose they were smaller than those in many other countries (e.g. the UK PEs were based on data from almost 93,000 households over a 15-year period). Importantly NZ household expenditure surveys do not record food price and purchase quantities (just expenditure) so price and quantity were derived from food price index data. This may have introduced some bias. If our method underestimated the true variability in food prices that consumers were exposed to, then we will have attributed consumption changes to ‘smaller-that-actual’ price changes, and thus overestimated PEs. However, if two products are close substitutes, then variations of the two prices tend to be correlated [27].

However our relatively higher PEs may be plausible for the following reasons: 1) New Zealand is a major food producing country, meaning consumers have good access to discounted food produce (e.g. fresh seasonal produce sold at discounted prices) and local farm- and home-grown produce/meat, i.e. substitute goods are accessible, increasing PEs; and 2) lower per capita income levels compared to other high-income countries means consumers are likely to be more responsive to food price changes. This is supported by an analysis of international data that ranked New Zealand tenth of 32 high-income countries based on ascending per capita real income levels (i.e. bottom one third) and reported food PE estimates for New Zealand that, for six of seven food groups examined, were 43−200% higher than the average for all high-income countries [25].

### Strengths and limitations

Our analyses comprise the most comprehensive food demand elasticity data available for New Zealand, which may be used as inputs in future work to forecast national food demand and supply and simulate effects of different government fiscal food policy options. Strengths include our use of food expenditure data from all major regions across a five-year period (2006 to 2010), which provided good price variability and enabled robust PE estimates for most food groups. We also provide measures of variability for own-PE and cross-PEs, indicating the precision and reliability of our estimates.

Significantly, ours is one of only a handful of studies that examined effects in food demand price elasticity by income level and ethnic group. One US study reported little difference in own-price elasticities between income groups for 12 food commodity categories [28], although a more recent analysis of US household dairy demand using 2007 scanner sales data found a significant effect of household income on dairy food purchases [29]. To the best of our knowledge only one other study has examined effects of ethnicity on food demand price elasticity, although ethnicity was not self-reported but was a proxy based on place of birth. Ulubasoglu et al reported that households with Australian-born heads had higher own-price elasticity for rice and more elastic demand for pork and dairy products compared to households whose heads were born overseas [26].

Nevertheless our analysis had limitations, specifically the absence of food price and quantity data in the HES survey dataset, and the relatively small population sample and short measurement period, which led to some unreliable PE values. Another limitation of our national expenditure datasets (in common with those for most countries) was aggregation of many nutritionally diverse foods within a single category, e.g. all milk, yoghurt and eggs were combined in one category, and all carbonated beverages in another. This made it impossible to assess the effects of price changes on close substitutes for many key foods e.g. full-fat versus reduced fat milk, or sugar-sweetened beverages versus sugar-free varieties. Understanding differences in price elasticity for close substitutes is important for food policy analyses concerned with reducing population saturated fat and sugar consumption. Economic theory suggests that PEs for close substitutes will be greater than observed in studies where all possible substitutes are combined in one category. However, existing observational datasets are extremely limited in this regard. Given the policy importance of these more targeted price changes, research to derive price elasticity values for close food substitutes is urgently required.

### Implications for policy

The effects of cigarette taxes on smoking prevalence demonstrate significant potential of pricing policies to modify behaviour [30]. Higher elasticity estimates suggest greater shifts in population purchases as prices change. From a public health perspective, more elastic demand for food is encouraging if change in demand is a priority. Our estimates suggest that a 10% tax on carbonated soft drinks could lead to a 13% decrease in population purchases of these products, whilst a 10% subsidy on fruit could lead to a 6.5% increase in purchases. Our most important finding is that low-income and Māori populations appear more sensitive to such price changes – similar to evidence that tobacco price elasticity is higher among low income populations.[17] Our results suggest that a 10% subsidy on vegetables would lead to a 6% increase in consumption by the highest income quintile, but a 11% increase in consumption among the lowest income quintile (Table 4).

## Conclusion

Although demand for food is relatively inelastic, the power of price changes to change consumer purchasing should not be underestimated given that effects accumulate across an entire population. The greater sensitivity of low-income and priority ethnic groups, such as Māori, to price changes suggests that targeted food pricing policies could alter the diets and nutritional health of these priority populations more than those of high-income and majority ethnic groups, thus making food pricing policy pro- health equity.
